# Beliefs around the causes of inequities and intergroup attitudes among health professional students before and after a course related to Indigenous Peoples and colonialism

**DOI:** 10.1186/s12909-023-04248-7

**Published:** 2023-04-22

**Authors:** Carolyn M. Melro, Kimberly Matheson, Amy Bombay

**Affiliations:** 1 Faculty of Health, Dalhousie University, 5869 University Avenue, P.O. Box 15000, Halifax, NS B3H 4R2 Canada; 2 Department of Neuroscience at Carleton University and the Culture & Gender Mental Health Research Chair, Ottawa, ON Canada; 3 The Royal’s Institute of Mental Health Research and Carleton University, Ottawa, ON Canada; 4 Department of Psychiatry and School of Nursing, Dalhousie University, Halifax, NS Canada

**Keywords:** Indigenous health, Cultural safety, Attitude change, Racism, Attribution bias

## Abstract

**Background:**

Addressing the Truth and Reconciliation Calls to Action on including anti-racism and cultural competency education is acknowledged within many health professional programs. However, little is known about the effects of a course related to Indigenous Peoples and colonialism on learners’ beliefs about the causes of inequities and intergroup attitudes.

**Methods:**

A total of 335 learners across three course cohorts (in 2019, 2020, 2022) of health professional programs (e.g., Dentistry/Dental Hygiene, Medicine, Nursing, and Pharmacy) at a Canadian university completed a survey prior to and 3 months following an educational intervention. The survey assessed gender, age, cultural identity, political ideology, and health professional program along with learners’ causal beliefs, blaming attitudes, support for social action and perceived professional responsibility to address inequities. Pre-post changes were assessed using mixed measures (Cohort x Time of measurement) analyses of variance, and demographic predictors of change were determined using multiple regression analyses. Pearson correlations were conducted to assess the relationship between the main outcome variables.

**Results:**

Only one cohort of learners reported change following the intervention, indicating greater awareness of the effects of historical aspects of colonialism on Indigenous Peoples inequities, but unexpectedly, expressed stronger blaming attitudes and less support for government social action and policy at the end of the course. When controlling for demographic variables, the strongest predictors of blaming attitudes towards Indigenous Peoples and lower support for government action were gender and health professional program. There was a negative correlation between historical factors and blaming attitudes suggesting that learners who were less willing to recognize the role of historical factors on health inequities were more likely to express blaming attitudes. Further, stronger support for government action or policies to address such inequities was associated with greater recognition of the causal effects of historical factors, and learners were less likely to express blaming attitudes.

**Conclusion:**

The findings with respect to blaming attitudes and lower support for government social action and policies suggested that educational interventions can have unexpected negative effects. As such, implementation of content to address the Truth and Reconciliation Commissions Calls to Action should be accompanied by rigorous research and evaluation that explore how attitudes are transformed across the health professional education journey to monitor intended and unintended effects.

**Supplementary Information:**

The online version contains supplementary material available at 10.1186/s12909-023-04248-7.

Globally, Indigenous Peoples experience health and social inequities compared to those who are non-Indigenous [1, 2], a fact known to most people living in settler colonial countries such as Canada [3]. Despite widespread knowledge about the existence of such inequities, many Canadians do not have basic knowledge of their shared colonial history and thus hold inaccurate beliefs about the causal factors contributing to Indigenous health and social issues [4, 5]. As described in the Truth and Reconciliation Commission (TRC) of Canada’s final report [6], the education system “has failed to teach this history” (p.286) and non-Indigenous Canadians have “little understanding of how the federal government contributed to this reality…” (p.286; 6). In line with Attribution Theory [7–9], without factual knowledge regarding the true root causes of inequities, non-Indigenous peoples may attribute the causes of group-based differences to intrinsic negative characteristics of Indigenous Peoples and underestimate the effects of external causes [3]. Such internal individual-level causal attributions for inequities held by advantaged group members who either do not know about or do not recognize the importance of structural determinants of well-being may lead those who are health professionals to provide second-class health care to Indigenous Peoples [4].

The good news is that educational interventions that teach about the social and structural determinants of group-based inequities in other contexts have been shown to increase external causal attributions [10] and reduce negative intergroup attitudes [11]. In line with this research, the TRC of Canada issued a call in its 2015 final report for the implementation of antiracist educational interventions and cultural competency training for healthcare professionals. These recommendations build on those of the Royal Commission on Aboriginal Peoples (1996; [12]) and call for explicit acknowledgement of causal links by including content focused on understanding colonialism and its effects on the health and social well-being of Indigenous Peoples. That said, positive outcomes of educational efforts aiming to improve intergroup relations have not always been evident [13], emphasizing the importance of evaluating such interventions to assess if they are having the intended effects [14]. Within the literature, few studies with practicing health professionals have explored the effect of educational interventions on general attitudes towards Indigenous Peoples [15, 16]. Despite the recent emergence of newly developed content about colonialism and its legacy being delivered in educational contexts since 2015, there is a lack of empirical evaluations assessing how learning about the harmful effects of colonialism is related to causal attributions for inequities and intergroup attitudes [17]. The current study explored the effect of a mandatory course that included content about colonialism among first year health professional students at an urban University. The analyses assessed changes in beliefs about the causes of health and social inequities (i.e., causal attributions) between non-Indigenous and Indigenous Peoples in Canada before and after learner’s complete the course. It was of interest to assess attitudinal changes in blame towards Indigenous Peoples for the inequities, and learners’ professional responsibility and support for actions to address inequities. As research has demonstrated that negative social attitudes have been linked with reduced perceived responsibility for addressing inequities [9, 18, 19] and support for actions [18, 20] to address these inequities.

## Educational Interventions related to Indigenous Peoples and colonialism

Euro-Caucasian settlers within Canada may deny or downplay the harms of colonialism and selectively focus on the 'positive benefits' [21], demonstrating an unwillingness to learn the true history of colonialism and its impacts on the health and social well-being of Indigenous Peoples. In this regard, the TRC final report [6] described how many non-Indigenous Canadians “hear about the problems faced by Indigenous communities, but they have almost no idea how these problems developed” (p.286). In line with this view, a 2019 national public opinion survey of Canadian adults demonstrated that almost half (42%) of non-Indigenous respondents viewed Indigenous Peoples to be of similar status as other cultural groups in Canada with more than double (29%) blaming Indigenous Peoples themselves for existing inequities compared to the 14% who blamed Canadian government policies [3]. Such views create an indifference towards colonial harms done and a justification of colonization as settler Canadians are motivated to believe systems are egalitarian. These egalitarian views in the health care system have become recognized as contributing to Indigenous health inequities [22–24].

A recent national public opinion poll, as previously described, found that older Canadians (age 40 and older) are twice as likely to blame Indigenous Peoples for the inequities they experience [3]. A possible explanation for this might be a greater awareness among younger people of the colonial trauma as a result of the TRCs Calls to Action that highlight the historical and social trauma endured by Indigenous Peoples. Such awareness might, in turn, diminish victim blaming attitudes, although there is considerable variation in such opinions among age groups. It was also found that Canadians living in the Prairies, and those who supported the Conservative political party were more likely to blame Indigenous Peoples for inequities [25]. In this regard, conservative ideologies have been associated with more victim-blaming attitudes generally [26–28].

Recognizing the pervasive continued racism and lack of knowledge regarding colonialism in Canada, learning about the legacies of colonialism appears to be a common learning objective in courses about Indigenous Peoples in Canada and beyond [16, 29]. Although some health professional programs had content related to Indigenous Peoples in place before the TRC call to action, there has been a rush for some health professional schools and professional licensing bodies in Canada to develop and deliver such content [30–33]. While the willingness is a positive step, it has unearthed considerable ambiguity and complexity in the development and evaluation of these interventions. Part of this complexity arises from the heterogeneity of conceptional models guiding course development (e.g., cultural safety, transformative learning theory), the diversity of specific course content, objectives, and approaches to evaluation [17, 34]. Despite differences across initiatives, a recent scoping review of 14 studies evaluating Indigenous health curriculum revealed that content was similar across interventions in Canada and other countries in their inclusion of learning objectives related to historical and political aspects of colonialism and social determinants of health [17], and most included related content, such as racism and privilege [17].

## Outcomes of educational interventions about Indigenous Peoples and colonialism: beliefs about the causes of Indigenous inequities

Although research has documented increased knowledge as a result of educational interventions about Indigenous Peoples [35–40], few have assessed changes in beliefs about the causes of Indigenous inequities. In the only study we are aware of, first year midwifery students in Australia were more likely to agree that “The state of Aboriginal health is mainly due to a lack of funding for health services” directly following a course, which the authors interpreted as reflecting “acknowledgement of structural factors at play in health status” (p.5; 40). That said, when these post-course responses among first-year students were compared with those of second- and third-year cohorts who had taken the course a year or two earlier, the first-year students were more likely to agree with this statement. The authors interpreted this as reflecting a decline in structural attributions over time since taking the course, which was supported by the fact that the second- and third-year students were less likely than the first-year students to agree that “The information I learned in this unit has changed my views on Aboriginal issues” (p.6; 40).

Although not specifically assessing causal attributions for inequities, beliefs related to understandings of colonialism and its effects on Indigenous Peoples have been assessed before and after educational interventions. For example, 98% of learners agreed or strongly agreed that the KAIROS Blanket Exercise “gave them a greater awareness of the impact colonization has had on Indigenous Peoples” (p. 1439; 35). In Australia, qualitative data from nursing students who completed a course dedicated to Indigenous history, culture and health revealed that some learners reported “increased knowledge and understanding of Indigenous history and the impact of past events and government policies on the health status of Indigenous Australians” (p. 464; 36). Learners in this study also described an enhanced awareness of the inherent challenges for Indigenous Australians related to the social determinants of health, and “developed their understanding of the effects of colonisation upon the health status of Indigenous peoples in the contemporary context.” (p. 465; 36).

## Outcomes of educational interventions about Indigenous Peoples and colonialism: attitudes towards Indigenous Peoples and their health inequities

Causal attributions about group-based inequities have been linked with intergroup attitudes among members of various advantaged groups [41]. However, very few educational interventions regarding colonialism and Indigenous Peoples have assessed intergroup attitudes before and afterwards [17]. In one such study, nursing students in Australia who took a course about Indigenous Peoples and colonialism reported less negative intergroup attitudes [36]; they were less likely to agree with statements such as ‘Land rights for Aborigines are just a way of them getting more than they deserve’ or ‘Aboriginal people get given more government money than they should’. Likewise, nursing students engaged in a cultural immersion service-learning experience in an isolated rural American Indian community reported more positive attitudes on items assessing blatant racist attitudes (e.g., I believe that Native Americans are inferior to Whites), modern racist attitudes (e.g., I don’t understand why Native American peoples blame all White people for this social misfortunes), and other related intergroup variables (e.g., I believe I know a lot about black [people’s customs]; I am comfortable talking to Native Americans; [42]).

Other potentially consequential attitudinal changes may be elicited by learning about colonialism and how it has contributed to health inequities. In this regard, an integrated Aboriginal health curriculum that included content about colonialism and its links with Indigenous well-being was associated with increased agreement among learners that they “had a social responsibility to work for change in Aboriginal health” [43]. Likewise, research in Australia indicated increased “intentions to improve current health inequities” among medical educators after a course that taught about colonialism [44]. However, another study that asked learners about their future work commitment (“I have a social responsibility to work for changes in Aboriginal health”) found no change after learning about colonialism, likely because the majority of students reported high perceived responsibility in the pre-course survey [40].

While conveying to learners accurate information about Indigenous Peoples may seem like a sensible and effective way of reducing false beliefs and negative attitudes [4, 5], there are various limitations to consider with this approach. Providing factual knowledge in a standalone short educational intervention – which is often how such information is delivered in health professional schools – may not always be successful in reducing negative intergroup attitudes when used in isolation [14], and even when immediate post-course attitudinal changes occur, they may not be sustained over time [38, 45, 46]. Such one-time interventions risk ‘essentializing’ populations, with the unintended effect of strengthening rather than debunking common myths and confirming stereotypes [14, 47–49]. An early study among medical students in Australia reported an increase in knowledge related to Indigenous Peoples after the course, but also an increased tendency to see all Aboriginals as the same [50]. Discussions about group inequities have been shown to elicit defensive [51] or resistant responses [22], and reduced attitudinal support for addressing inequities [52, 53]. Such potentially diverging outcomes emphasize the importance of evaluating educational interventions that include content about the social determinants of health and group-based inequities to ensure that they are not having the unintended effects of making racist beliefs and attitudes worse.

## The current study

Given the urgency of addressing discriminatory healthcare beliefs and practices through health professional education that increases knowledge and positive intergroup attitudes, there is a need to assess the intended and unintended consequences of training related to Indigenous Peoples and their well-being. To date, little research has explored the effects of educational interventions on health professional learners’ causal beliefs about health and social inequities facing Indigenous Peoples, attitudes that blame Indigenous Peoples for such inequities, as well as learners’ sense of responsibility and support for actions to address inequities. This evaluation assessed the changes of beliefs and attitudes following a course for first-year health professional students that teaches about colonialism and its legacy. It was anticipated that the intervention would lead to an increased endorsement of structural causal beliefs (i.e., recognition of historical and ongoing colonialism), lower victim-blaming attitudes, an increase in perceived responsibility, and support for action to address inequities, among learners from a range of health professional programs. In addition, we will examine the role of learner age and political views on influencing training outcomes.

## Methods

This study utilized the opportunity afforded by the introduction of mandatory content for first-year health professional students at an urban university. A module-based course was introduced in 2019 with content presented both in-person and online including readings, group work, case scenarios, and lectures with faculty, Elders, and facilitators over four weeks. In 2022, content building on Indigenous knowledge to create better health care and outcomes was added and the course was extended to six weeks. The following content was consistent across three course offerings (2019, 2020, and 2022): Indigenous People, History & Health; Indigenous Peoples’ Perspectives on Health Issues; Clinical Strategies for Indigenous Health; and, Learning How to Integrate New Knowledge into Practice. The course was informed by the concept of Cultural Safety and developed by Indigenous and non-Indigenous faculty members in partnership with Elders, Knowledge Keepers, and community members. In addition to content on culturally safe health care, the course provided an overview of national and regional Indigenous health and social outcomes facing Indigenous Peoples, and addressed two learning objectives related to colonialism and its legacy:

1. “Acquire knowledge about the Indigenous People who live in Canada, where they live and important historical events that have affected their health and wellbeing; and,
2. Understand the current day impact of historical injustices, racism and how policy and landmark decisions (Indian Act, Indian Residential Schools, Sixties Scoop, United Nations Declaration of the Rights of Indigenous Peoples, Indian Day Schools, etc.) can and have shaped health care systems”.

### Participants and procedures

A self-report questionnaire was administered via email to a total of 1208 first-year health professional learners enrolled in the mandatory course, *Introduction to Culturally Safe Care for Indigenous Peoples* at baseline (before or at the start of course) over the three years. The survey advertisement was posted on the course homepage. Although it would have been preferable for students to complete the questionnaire during in-class time to garner a higher response rate, this was not permitted as the course evaluation was voluntary for learners. This evaluation study was conducted in accordance with the Tri-Council Policy Statement and exempt from the University research ethics review as it was considered program evaluation [54, 55]. Informed consent was provided by all participants for both baseline and post-course surveys. The study was not required to follow Indigenous research principles such as Ownership, Control, Access and Possession (OCAP) as the participants in the study are mostly non-Indigenous health professional learners. If students self-identified as Indigenous, a warning was provided on the nature of the questions, and they were asked if they wished to proceed. The survey took approximately 20 to 30 min to complete. Only learners who participated in the baseline survey were invited to participate in the post-course survey. Those who agreed to be contacted were sent an email 3-months following the course completion, with reminder emails sent to encourage participation. A follow-up period of 3-months was chosen given the mixed findings of pre- and post-course evaluations immediately after the educational intervention. As such 3-months was chosen as it aligns with the end of the academic term. All learners were provided with the contact information for the student wellness centre and crisis response if they experienced any distress in participating in the study. Respondents in the 2020 and 2022 cohorts received a modest monetary incentive ($5) for completing the post-course survey.

### Measures

Participants were asked to create a unique identifier when completing the baseline survey in order to link survey responses across the two measurement time points. Demographic variables, including health professional program, age, gender identity, Indigenous identity, and political views, were captured at baseline. Socio-demographic questions were measured using open-ended questions and coded for man and woman; and Indigenous and non-Indigenous, respectively. Political views were elicited with the following question: *What political group do you support?* with the response options: New Democratic Party, Green Party, Conservative, Liberal, None and Other. From least to most socially conservative, political parties in Canada would be arranged Green, NDP, Liberal, Conservative. For all remaining items, participants responded using a seven-point Likert-type scale ranging from 1 (Strongly Disagree) to 7 (Strongly Agree) (along with a ‘do not know’ option that was coded as missing). In all instances, mean scores were calculated, with relevant items reverse scored. Cronbach’s alphas were calculated to assess the internal consistency with each cohort, pre- and post- Cronbach’s α are reported in this paper. Many demonstrated strong internal consistency, although not all measures had strong Cronbach’ alphas. The full questionnaires are appended as an Additional File (See additional file 1).

Beliefs and attitudes were assessed using slightly revised questions adapted from a previous unpublished study by our team assessing training about colonialism in another context by (Melro CM, Bombay A: Summary report: Indigenous Service Canada’s Pilot Program on Understanding the root causes of health and social inequities between Indigenous and non-Indigenous (Settler) people in Canada, unpublished). These questions were created with input from an Indigenous Advisory Committee comprised of Indigenous individuals with expertise in Indigenous health.

To assess the component structure of the measure created to assess causal beliefs about Indigenous Peoples’ inequities, the 12 items were subjected to a principal component analysis with varimax rotation. For the purpose of the paper, we report the findings of the principal component analysis for the pre- and post- 2022 cohort. Two components had eigenvalues greater than 1 (6.64 and 1.28, respectively) accounting for 66.04% of the variance. Subscale scores were created by averaging unit-weighted responses to items with loadings greater than 0.45; if an item met this criterion on both components, they were included in the subscale with the highest loading (See Table 1) Based on the items loading highly onto each of the components, the first was labeled *historical aspects of colonization as a cause of health/social gaps* (6 items), whereas the second subscale appeared to reflect *ongoing effects of colonialism* (6-items).

**Table 1:** Rotated component loadings of individual difference scale scores for Causal Beliefs about Indigenous Inequities

*Item*	*Component 1*	*Component 2*
The negative effects of the Residential School System are a significant contributor to the health and social gaps that exist between Indigenous and non-Indigenous peoples today	**.780**	
Indian Residential Schools were in the distant past so they probably don’t play a huge role in the health/social gaps that exist today between Indigenous and non-Indigenous Canadians (R)	**.823**	
The negative effects of the Residential School System have been transferred from one generation to the next and contribute to ongoing gaps in health/social outcomes between Indigenous and non-Indigenous peoples	**.828**	
Numerous policies put into place through the Indian Act over generations have contributed to the present-day health disparities affecting Indigenous Peoples	**.836**	
It is unlikely that the residential school system has negatively affected the well-being of the children and grandchildren of those who attended these schools. (R)	**.882**	
There is no relationship between the social determinants of health (e.g., housing, income, education) and the historical and on-going health and social gaps between Indigenous and non-Indigenous peoples (R)	**.619**	.456
Indigenous Peoples in some contexts do not receive equitable health services which contribute to ongoing health disparities		**.517**
Indigenous Peoples in Canada receive the same amount or more funding for social and health services relative to non-Indigenous Canadians (R)		**.848**
Indigenous People in Canada have equal or more access to government-provided health care. (R)		**.800**
Differences between Indigenous and non-Indigenous Peoples in key social determinants of health such as income and education play a significant role in contributing to health and social inequities between these groups	.482	**.574**
Certain on-going government policies related to the provision of social services contribute to the ongoing health inequities facing the Indigenous Peoples in Canada	.490	**.609**
The long-term effects of the residential school system have been over-exaggerated in the media and/or society in general. (R)	.552	**.611**

The Cronbach’s α are reported for each of the three cohorts given changes made to the survey items in each iteration (See Table 1). It is noted that the Cronbach’s α coefficient in the 2020 cohort demonstrated a slight decline over time, although still within an acceptable range. It is possible that nuances of understanding derived from the training gave rise to more inter-item variability in the responses of this group following the intervention.

Blaming attitudes towards Indigenous Peoples were assessed using 8-items (pre- Cronbach’s α = 0.835, post– Cronbach’s α = 0.889) adapted from Gomez and Wilson (2006; [24]). Participants responded to items such as ‘Most of the health and social problems of Indigenous people are brought on by themselves’; ‘Indigenous Peoples in Canada face unique historical, cultural, and social determinants of health associated with colonization that has affected their well-being’; and, ‘Indigenous Peoples in Canada face unique historical, cultural, and social determinants of health associated with colonization that has affected their well-being’.

Perceived responsibility as a future healthcare provider was assessed on 4-items that were developed for this study (e.g., ‘I have a social responsibility to work with Indigenous Peoples to improve their social and health conditions’ and ‘My mandate as a health professional does not include attention to the unique factors that may affect Indigenous Peoples and is instead focused on providing equal care to all patients’ (R); pre- Cronbach’s = 0.890, post– Cronbach’s α = 0.867). Only one component was extracted with an eigenvalue of 2.434, accounting for 60.84% of the variance. Nine items were developed for this study that assessed Support for actions to reduce inequities, including the following example items: ‘The federal government is spending too much on improving the living conditions of Indigenous Peoples’ (R); ‘Social policies for Indigenous Peoples such as affirmative action should not be instituted because they discriminate unfairly against others’ (R), and ‘Indigenous Peoples should be treated like all Canadians and should not have any special benefits or rights to land or to hunt/fish’ (pre- Cronbach’s α = 0.884, post- Cronbach’s α = 0.768). The nine-items were subjected to a principal component analysis with varimax rotation. Two components ahd eigenvalues greater than 1 (5.53 and 1.07, respectively). However, all of the items had loadings greater than,45 on the first component. Given this, average scores were based on consideration of only one component.

### Analysis

Statistical analyses were conducted using the Statistical Package for the Social Science (SPSS) version 27.0 [56]. Data were cleaned, with outlier scores (i.e., defined as 3 standard deviations above or below the mean score) removed. Data analysis was conducted in four phases. First, descriptive statistics (e.g., frequencies, means, variance) were provided for each of the measures, as well as associations with demographic characteristics. Second, mixed measures analyses of variance (ANOVAs) were conducted to assess changes in response to exposure to the course (baseline vs. 3 months post course) in each of the three course administrations (2019 vs. 2020 vs. 2022). Third, demographic predictors (e.g., health professional program, age, gender) of pre-post differences were assessed using multiple linear regression. Lastly, Pearson correlations were conducted with all three cohorts to identify relationships among outcome variables.

## Results

### Demographic characteristics of learner population

In total, 335 first-year learners (*M* age = 23.6 years, *SD* = 4.44) responded to the pre- and post-survey (*n* = 76 in 2019; *n* = 154 in 2020; *n* = 105 in 2022). As seen in Table 2, the majority of learners identified as women and as non-Indigenous. Political views were only measured in the 2020 and 2022 cohorts, with the majority expressing left-leaning views (the New Democratic Party). A series of chi-square independence tests indicated that health professional program, χ^2^ (6, *N* = 304) = 74.33, *p* < 0.001, gender, χ^2^ (4, *N* = 304) = 110.86, *p* < 0.001, Indigenous identity χ^2^ (2, *N* = 299) = 7.19,* p* = 0.028, and political views, χ^2^ (6, *N* = 238) = 21.48, *p* = 0.002), varied across the three cohorts (see Table 2), as did age, *F*(1, 301) = 4.74, *p* = 0.030. The 2020 cohort had the highest representation of Indigenous students, and expressed more liberal views (35.8%) compared to the 2022 cohort which was strongest in support of the New Democratic Party (37.5%). The 2019 cohort was slightly older than the 2022 cohort, but neither differed in mean age from the 2020 cohort.

**Table 2:** Demographic characteristics of study sample

	**2019** **(*n* = 76)**	**2020** **(*n* = 154)**	**2022** **(*n* = 105)**
**Health professional programs:**			
Medicine	26 (34.2%)	44 (32.6%)	24 (22.9%)
Nursing	28 (38.8%)	73 (54.1%)	46 (43.8%)
Dentistry/Dental Hygiene	11 (14.5%)	17 (12.6%)	20 (19.0%)
Pharmacy	N/A	N/A	15 (14.3.%)
**Gender:**			
Women	47 (71.2%)	106 (79.1%)	87 (83.7%)
Men	19 (28.8%)	28 (20.9%)	15 (14.4%)
Non-Binary	1 (1.3%)	N/A	2 (1.9%)
**Mean Age**	24.61 (19 to 39)	23.69 (19 to 44)	23.08 (19 to 40)
**Indigenous identity:**			
Non-Indigenous	60 (90.9%)	119 (88.8%)	94 (89.5%)
Indigenous	6 (9.1%)	15 (11.2%)	11 (10.5%)
**Political views:**	^a^Missing data		
Conservatives		4 (3.0%)	12 (11.5%)
Liberals		48 (35.6)	21 (20.2%)
New Democrat Party		39 (28.9%)	39 (37.5%)
Green Party		23 (17%)	9 (8.7%)
None		N/A	21 (20.2%)
Other		18 (13.3%)	2 (1.9%)

^a^In 2019, respondents were asked how they would rate their political attitudes on a sliding scale between liberal (left wing) to conservative (right wing). We did not include these data in our analysis

### Intervention effects

**Fig. 1:**
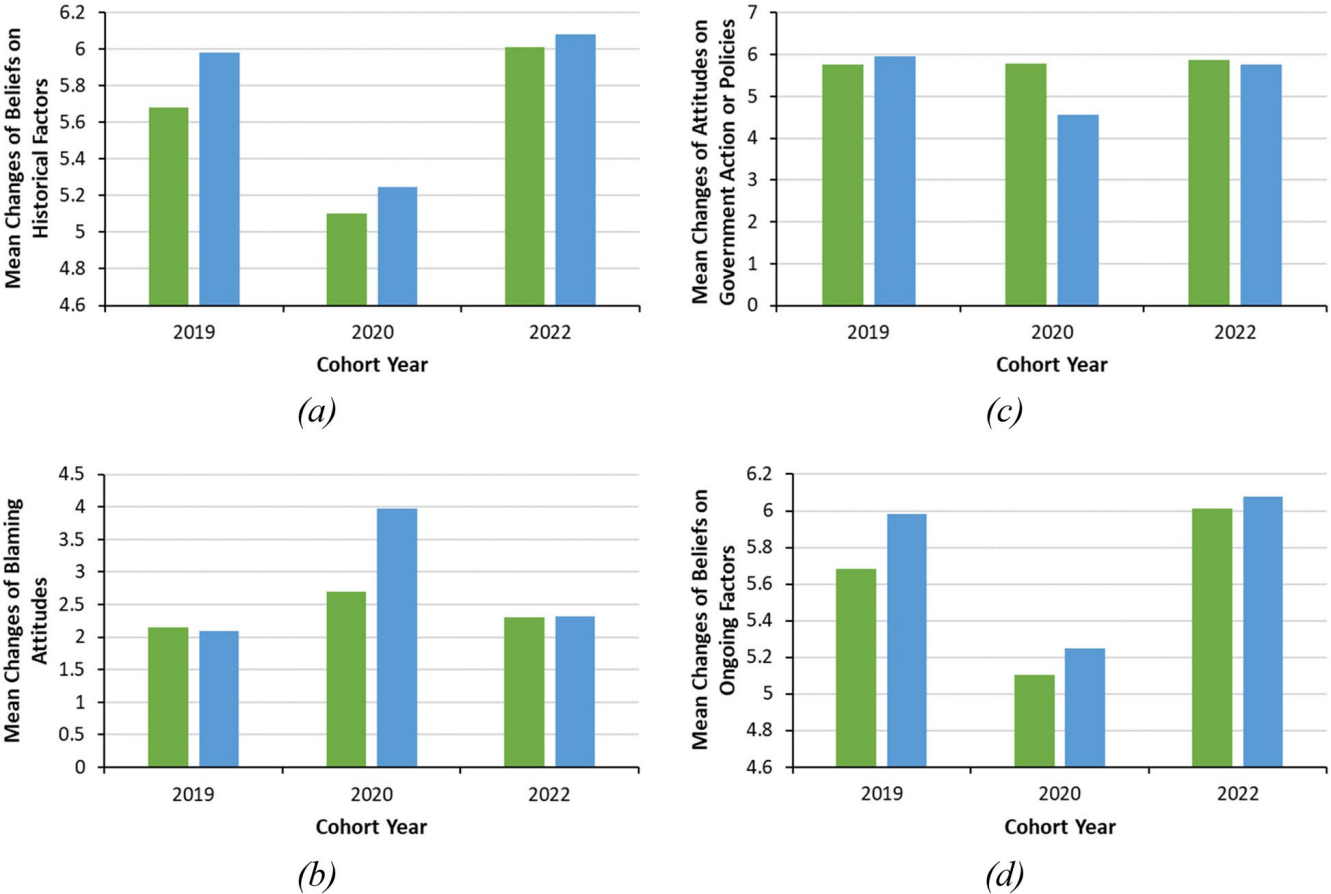
Mean levels of agreement on (**a**) Causal beliefs—historical factors, (**b**) blaming attitudes, (**c**) support for government action or policies, and (**d**) Causal beliefs—ongoing factors at baseline and 3 months following the educational interventions. Note: Pre-Course – green bar; Post-Course – blue bar. Items were measured on a seven-point Likert-type scale with 1 ‘strongly disagree’ and 7 ‘strongly agree’

To determine whether causal beliefs, blaming attitudes, professional responsibility to address inequities and support for government action or policies changed as a function of the educational intervention, a series of 2 (pre- and post-course) X 3 (cohort) mixed measures analyses of variance (ANOVAs) was conducted. There were significant interactions suggesting that the intervention differentially affected the three cohorts in terms of learners’ causal beliefs regarding historical factors of colonialism, *F* (2,273) = 2.79, *p* < 0.001, η^*2*^ = 0.020, blaming attitudes, *F* (2, 276) = 102.09, *p* < 0.001, η^*2*^= 0.425, and support for government action and policies to address inequities, *F* (2,273) = 83.79, *p* < 0.001, η^*2*^ = 0.380. Simple main effects comparing the pre-post responses for each of the three cohorts showed that, following the intervention, learners in the 2020 cohort were *more* likely to believe historical factors contributed to present day inequities (Fig. 1a) and to express blaming attitudes (Fig. 1b) and were *less* likely to support government action and policies to address inequities (Fig. 1c); these differences were not significant among learners in the 2019 and 2022 cohorts, although they demonstrated a similar trend to the 2020 cohort. This interaction was not significant in relation to causal beliefs regarding ongoing factors, *F* (2, 273) = 1.43, *p* = 0.240, η^*2*^ = 0.020, or professional responsibility to address inequities, *F* (1, 223) = 3.07, *p* = 0.081,η^*2*^ = 0.014, nor were there significant main effects for the intervention itself on professional responsibility to address inequities, *F* (1, 223) = 0.001, *p* = 0.971, η^*2*^ = 0.000*.* However, there was a main effect for cohort for ongoing factors, *F* (2, 274) = 47.14, *p* < 0.001,η^*2*^ = 0.256. As seen in Fig. 1d, beliefs that ongoing colonialism contributed to health inequities for Indigenous Peoples were less likely to be endorsed by learners in the 2020 cohort compared to learners in the 2019 and 2022 cohorts, which did not differ from each other (with Tukey adjustment for familywise error at *p* < 0.05). There was no significant main effect for cohort on taking professional responsibility to address inequities, F (1, 223) = 0.001, *p* = 0.971.

### Correlations among outcome variables

Pearson correlation coefficients were conducted to assess the linear relationships among causal beliefs regarding the role of historical factors, blaming attitudes and support for social action or policies. There was a negative correlation between historical factors and blaming attitudes, *r* (283) = -0.36, *p* < 0.001, suggesting that learners who were less willing to recognize the role of historical factors on health inequities were more likely to express blaming attitudes. Moreover, stronger support for government action or policies to address such inequities was associated with greater recognition of the causal effects of historical factors, *r* (282) = 0.52, *p* < 0.001, and lower inclination to express blaming attitudes, *r* (282) = -0.88, *p* < 0.001.

### Demographic predictors of responses to the intervention

Multiple regression analyses were conducted to assess the demographic features that predict learners’ responsiveness to the educational intervention. Based on the above reported interaction effects, each outcome variable at Time 2 (Causal Beliefs – Historical Factors, Blaming Attitudes, Support for Government Action and Policies) was regressed onto levels at time 1 on the first step, followed by age (continuous), gender (male coded 0 vs. female coded 1), and Indigenous identity (non-Indigenous coded 0 vs. Indigenous coded 1) on the second step, the null variables representing health professional program on the third, and finally the null variables representing political views on the last step. By entering the variables in blocks, we were able to evaluate the added variance accounted for in the outcome variables. Regression coefficients in the final (4^th^) step were the basis of our interpretations of the patterns of relationships.

The multiple linear regression model demonstrated that learners who had awareness of historical factors prior to the intervention were more likely to recognize historical factors as being an important causal belief as to why Indigenous Peoples are more likely to experience inequities following the intervention, *R*^*2*^ = 0.464, *F*(1, 214) = 184.88, *p* < 0.001. However, none of the demographic variables predicted such beliefs (see Table 2).

The extent to which learners expressed blaming attitudes following the educational intervention was, not surprisingly, greater among those who held such attitudes at the outset, *R*^*2*^ = 0.146, *F*(1, 219) = 38.16, *p* < 0.001. As a whole, demographic variables accounted for an additional 35.2% of the variability of post-intervention blaming attitudes. In particular, as seen in Table 3, males (*M*_*adj*_ = 3.65, *se* = 0.069) were more likely than females (*M*_*adj*_ = 2.53, *se* = 0.065) to continue to express blaming attitudes following the intervention (means are adjusted for baseline scores). In addition, the intervention appeared to be differentially effective depending on the program learners were registered in, *R*^*2*^_*change*_ = 0.012, *F*(3, 210) = 6.11, *p* = 0.001 (Table 3). Post hoc pairwise comparisons (with Tukey adjusted *p*-values to maintain family-wise α less than 0.05) indicated, following the intervention, the dentistry and dental hygiene learners (*M*_*adj*_ = 3.58, *se* = 0.090) were significantly *more* likely to express blaming attitudes than those in medicine (*M*_*adj*_ = 2.71, *se* = 0.111), nursing (*M*_*adj*_ = 2.87, *se* = 0.083), or pharmacy (*M*_*adj*_ = 2.19, *se* = 0.252). None of the other demographic variables was a significant predictor of changes in blaming attitudes.

**Table 3:** Final step unstandardized linear regression coefficients predicting beliefs that historical factors caused inequities, blaming attitudes, and support for social action or policies

Causal Beliefs -Historical Factors				
F (3, 205) = 19.79, *p* < .001, Adjusted R^2^ = .466				
**Predictors**	**β**	**SE**	***p***	**Zero order correlation**
Pre-course	.657	.047	.001	.681
Age	.050	.011	.348	.209
Gender	-.114	.113	.070	-.037
Indigenous Identity	.005	.143	.916	.916
Political Views^a^				
*Conservatives*	-.067	.192	.202	-.130
*Liberal*	-.046	.143	.676	-.002
*Green*	-.023	.108	.397	.052
Health Professional Program^a^				
*Medicine*	-.039	.133	.491	-.045
*Dentistry*	-.136	.121	.037	-.085
*Pharmacy*	.047	.202	.381	.085
Political Views^b^				
*Conservatives*	-.067	.192	.202	-.130
*Liberal*	-.046	.143	.676	-.002
*Green*	-.023	.108	.397	.052
**Blaming attitudes**				
F (10, 210) = 23.02, p < .001, Adjusted R^2^ = .500				
**Predictors**	**β**	**SE**	***p***	***Zero order correlation***
Pre-course	.331	.065	.001	.389
Age	.040	.011	.427	.034
Gender	-.431	.113	.001	-.601
Indigenous Identity	.038	.149	.434	.019
Health Professional Program^a^				
*Medicine*	-.090	.136	.094	-.144
*Dentistry*	.114	.126	.072	.433
*Pharmacy*	-.151	.216	.003	-.313
Political Views^b^				
*Conservatives*	.018	.194	.713	-.056
*Liberal*	.078	.149	.136	.097
*Green*	.107	.111	.041	.145
**Support for government social action or policies**				
F (10, 206) = 19.08, *p* < .001, Adjusted R^2^ = .456				
**Predictors**	**β**	**SE**	***p***	***Zero order correlation***
Pre-course	.426	.053	.001	.423
Age	.027	.011	.613	.044
Gender	.450	.113	< .001	.508
Indigenous Identity	-.004	.145	.932	.047
Health Professional Program^a^				
*Medicine*	.065	.132	.258	.093
*Dentistry*	-.043	.122	.514	-.350
* Pharmacy*	.156	.210	.004	.282
Political Views^b^				
*Conservatives*	-.032	.194	.563	-.069
*Liberal*	-.002	.143	.971	-.037
*Green*	-.039	.109	.481	-.086

*SE* Standard Error

Coefficients are taken from the final step of the regression models

^a^Three null variables were created to represent Health Professional Program with endorsements for each of Medicine, Dentistry, and Pharmacy coded ‘1’ on the respective variable, and enrollment in Nursing coded as ‘0’ on all three variables

^b^Three null variables were created to represent Political Views with endorsements for each of Conservative, Liberal, and Green parties coded ‘1’ on the respective variable, and endorsement of the New Democratic party coded as ‘0’ on all three variables

Similarly, support for government action and policies was greater among those who expressed more supportive views at the onset of the course, *R*^*2*^ = 0.179, *F*(1, 215) = 46.93, *p* < 0.001. Once again, as seen in Table 4, participant gender was significant with females (*M* = 5.68, *se* = 0.062) being more likely to express greater support government social action and policies following the intervention (i.e., controlling for pre-intervention levels) than males (*M* = 4.74, *se* = 0.065). The intervention also appeared to be more effective for learners in different programs, *R*^*2*^_*change*_ = 0.027, *F*(3, 209) = 27.435, *p* = 0.001 (Table 4). Post hoc pairwise comparisons indicated support for government policies was lower among dentistry and dental hygiene learners (*M* = 4.79, *se* = 0.083) than among medicine (*M* = 5.57, *se* = 0.102), nursing (*M* = 5.35, *se* = 0.079), and pharmacy (*M* = 6.04, *se* = 0.235) at *p* < 0.001. None of the other demographic variables was a significant predictor of changes in blaming attitudes.

**Table 4:** Cronbach’s α for Causal beliefs about Indigenous Peoples inequities

Causal Beliefs – Historical aspects of colonization as a cause of health/social gaps					
2019		2020		2022	
Pre	Post	Pre	Post	Pre	Post
.671	.667	.947	.864	.892	.909
Causal Beliefs – Ongoing aspects of colonialism					
2019		2020		2022	
Pre	Post	Pre	Post	Pre	Post
.859	.835	.548	.688	.819	.838

## Discussion

The primary aim of this study was to examine whether completing the *Introduction to Culturally Safe Care for Indigenous Peoples* course influenced health professional learners’ causal beliefs about Indigenous inequities, intergroup attitudes, and responsibility to address inequities from baseline to 3-months post-training. Our findings indicate that there was a mix of intended and unintended outcomes found, but these were only observed in the 2020 cohort. Although these outcomes were unchanged after the course for the 2019 and 2022 cohort, learners in the 2020, learners were more likely to believe historical factors contributed to present-day inequities at the end of the course. However, in this same cohort, unexpectedly, blaming attitudes were more likely to be expressed towards Indigenous Peoples and learners were less likely to support government action and policies to address inequities. Clearly, there was something unique about the 2020 cohort that may have contributed to their reactions. Indeed, they were less likely from the outset to indicate beliefs that on-going factors of colonialism might be causing health inequities. This lack of recognition may reflect a lack of knowledge (that did not change in response to the intervention) or may have elicited reactance to efforts to bring about changed understandings. That said, the reason for these different outcomes in the 2020 cohort are unclear and may warrant more consideration of external or environmental factors that may influence course outcomes. It may also be possible that some subtle differences in the way the course was delivered could have affected these outcomes. That said, the reason for these different outcomes in the 2020 cohort are unclear and may warrant more consideration of external or environmental factors that may influence course outcomes. It may also be possible that some subtle differences in the way the course was delivered could have affected these outcomes. Potential external or environment factors that occurred in 2020 that could contribute to these findings is the increased awareness, globally and nationally, of social injustices towards outgroup members (e.g., Black Lives Matter Protests, the Mi’kmaw fishery dispute on Treaty Rights, and the death of Joyce Echaquan) all occurring during the COVID-19 pandemic.

Gender differences on outcome variables were found within our study. For instance, the results of this study suggest that male learners in our sample are more likely to express blaming attitudes towards Indigenous Peoples following the intervention. This finding is consistent with findings from a national public opinion survey of non-Indigenous Canadians that demonstrated that men were twice as likely to blame Indigenous Peoples themselves for the inequality they face compared to woman [3]. Similarly, gender differences or political views did not differ based on individual or structural attributions [10, 57], this aligns with the findings of our study. Our study also suggest that women were more likely to express greater support for government social action and policies following the intervention. Given, that woman were less likely to demonstrate blaming attitudes post-course this finding aligns with other research [58–62].

Interestingly within our sample dentistry and dental hygiene learners were significantly *more* likely to express blaming attitudes and were *less likely* to indicate support for government policies compared to those in the other health professional programs (i.e., medicine, nursing, or pharmacy). It could be hypothesized that dentistry and dental hygiene programs lack broad educational interventions on social and structural determinants of health in comparison to other health professional programs.

Not surprisingly, in our study, those who expressed blaming attitudes at the end of the course were less likely to support government action to address inequities. This is consistent with research that has found that the public is less likely to support social action and policies to address inequities when they attribute blame to individual victims rather than the systems or social determinants of health that continue to perpetuate inequities [58–62]. Likewise, recognizing the role of historical factors in health inequities and blaming attitudes were associated with support for government social action and policies in the present study. Attributions of causes of social conditions to individual or social factors are influenced by personal experience and socialization to norms and values from the groups in which individuals identify (e.g., cultural identity, socioeconomic status, political views) and related perceptions of deservingness [62]. It has been suggested that attributions for events are grounded in individuals’ social identities and are often relied on to justify our social worlds and maintain inequities [63–65], and challenges to those beliefs might elicit affective reactions (e.g., denial and defensiveness; [37, 38, 45, 46, 65]). Further research should explore the relationship between learners’ affective reactions (e.g., defensiveness) to educational interventions and their beliefs (e.g., system justification and causal beliefs), blaming attitudes, and support for addressing inequities. Motivational processes, particularly but not solely among advantaged groups (as our findings did not indicate differences between Indigenous and non-Indigenous learners), may evoke defensive reactions to justify historical and ongoing colonialism that are the root causes of Indigenous Peoples’ outcomes by viewing existing social, political, and economic systems as fair and legitimate [63]. System-justifying ideologies in which people are motivated to justify and rationalize the way things are by viewing existing social, political and economic (e.g., distal structural determinants of health) systems as fair and legitimate [63], may influence how learners engage with such content. These motivational processes may reflect cognitive biases (in attributing causality), enabling dominant group members to justify the neglect, powerlessness, and social injustices that are the root causes of social outcomes [63].

Possible reasonings for the incline in some learners’ blaming attitudes post-course can be that learners might have romanticized notions of Indigenous Peoples and experience dissonance when they learn the reality of the historical misconceptions. This may have an unintended effect of evoking blaming attitudes and ingraining explanatory stereotypes as to why Indigenous Peoples experience inequities [66]. The timing of the post-course survey in the Spring of 2020 (May to June) aligned with the global protests such as Black Lives Matter. Such media coverage and heightened awareness of social injustices towards out-group members may have affected learners’ responses or resistance to course material. For instance, after first year students of the Bachelor of Health Sciences completed a Maori Health Issues and Opportunities course, learners demonstrated factual learning about colonization, but continued to interpret the content through a deficit lens suggesting that after learning about the impact of colonization students were observed to perpetuate ‘charitable racism’ [67]. This finding demonstrates the need for careful pedagogical consideration of how the impact of colonization is taught to health professional learners, ensuring that they are critically reflecting on systems of oppression and privilege. Further, it is a reminder to educators that while seeking to present a clear and coherent narrative there is the risk of oversimplifying and essentializing Indigenous Peoples that can lead to individual explanations versus structural and social explanations of causes of inequities that contribute to blaming attitudes. As such, targeted interventions should be intentionally developed to challenge ingrained causal beliefs and attitudes and continue to be monitored for both intended and unintended changes to inform the development of educational interventions.

## Limitations and future opportunities

In addition to the strengths of this study, there are limitations that warrant discussion. As is common to self-report studies, although we employed confidentiality measures to protect the participants' identity, a social desirability response bias may have occurred, particularly given that the topics covered in the course were potentially sensitive and polarizing. There was likely a selection bias of those who participated in the course evaluation compared to those who did not, as participation was optional. Finally, as within any closed-survey format, participants were limited in their responses and unable to provide their own narratives. As such, we may have missed nuance and additional information that could have been helpful in understanding some of the beliefs and attitudes expressed. An iterative process to survey development was followed to create the measure of *causal attribution beliefs about the causes of Indigenous health and social inequities* instrument, lending it some validity to tapping into key perspectives. However, given the novelty of this measure each iteration of the survey (and hence cohort) had small adjustments to item wording (See Additional File 2). The items included in the supplementary material is the latest version used with the 2022 cohort. Future research would benefit from a more rigorous application of common measures and experimental design including a comparison group to determine the effect of the educational intervention on dispelling false beliefs and challenging negative attitudes towards Indigenous Peoples in health professional programs. This includes robust scale development and factor analysis of a novel measure. A longitudinal study would help to explore which kinds of content, pedagogical approaches, and how much exposure to content about Indigenous Peoples is required for change and maintenance of such changes in beliefs, attitudes, and support for change. A longitudinal study might help identify curriculum or system barriers to changing beliefs, attitudes and behaviours.

## Conclusion

Despite recommendations by health professional schools and governing license bodies to embed Indigenous cultural competency and anti-racism education in *all* university programs [6], the question remains as to what the intended and unintended effects of Indigenous educational interventions are on learners’ beliefs, attitudes, and perceived responsibility. This study found no effects in two learner cohorts, and an unintended effect in one cohort, wherein the learners’ blaming attitudes, sense of professional responsibility, and support for government social action and policy *worsened* at the end of the course. Implementation of such content should be accompanied by rigorous research and evaluation that explore how attitudes are transformed across the health professional education journey. Particularly, we call for more realist evaluations in which researchers advance the course-based literature from merely does it work to what works, for whom and in what contexts.

## Supplementary Information

**Additional file 1.**

**Additional file 2.** Development of the Causal beliefs aboutIndigenous Peoples inequities Measure.

## Data Availability

The datasets generated and/or analysed during the current study are not publicly available due no ethics approval was sought from participants to share raw data in the design of the project but are available from the corresponding author on reasonable request.
