# Supplementary material for: Beliefs around the causes of inequities and intergroup attitudes among health professional students before and after a course related to Indigenous Peoples and colonialism
Source: BMC Med Educ. 2023 Apr 22;23:277. doi: 10.1186/s12909-023-04248-7 (PMC10121421; doi:10.1186/s12909-023-04248-7)
Supplement: Supplementary file 1 — Additional file 1. [file 12909_2023_4248_MOESM1_ESM.docx]

Additional File 1

**Pre-course survey**

**For us to be able to connect your responses on the anonymous pre-course and post-course surveys, we ask you to create a USERID. This is your initials, and the last digit of your birth month, date, and year (e.g., AM298, Name - Angela MacDougal Birthday – February 9^th^, 1998).**

**USERID: ______________**

**Post course survey**

**For us to be able to connect your responses on the anonymous pre-course and post-course surveys, we asked you to create a USERID at the beginning of the last survey. This is your initials, and the last digit of your birth month, date, and year (e.g., AM298, Name - Angela MacDougal Birthday – February 9th, 1998).**

**USERID: ______________**

Demographic information

1. Gender and gender-expression exists on a spectrum. To be as inclusive as possible, we have left an open space to identify your own gender. Some examples of gender include, but are not limited to, female, male, trans*, two-spirit, and agender.

Gender: __________________________________

1. Age: ________
2. Cultural Identity: Your cultural identity is your self-defined sense of belonging to a group – which could include race, ethnicity, nationality, religion, province, locality – or any social group that has its own distinct culture.

Please list/describe which ethno-racial-cultural group(s) you identify with and/or that you consider to be part of your cultural identity. List as many as apply. Examples include Scottish, Mi'kmaq, Italian, African Nova Scotian, Lebanese, Inuit, Irish, Acadian, Jewish, English, Canadian, Quebecois, Chinese-Canadian, Colombian, Newfoundlander, German, etc.

Specify: ____________________________________________________________________

1. Does your cultural identity include any of the three Indigenous groups in Canada? And/or identification with a specific Indigenous community?

- No, I do not have any Indigenous heritage (i.e., First Nations, Metis, Inuit)
- First Nations
- Métis
- Inuit

Please feel free to elaborate on your answer:

__________________________________________________________________________________________________________________________________________________________________________________________________________________________________________

1. Not surprisingly, one’s political views are often associated with their views on various social issues. What political group do you support?

- NDP
- Green Party
- Conservative
- Liberal
- None

Causal Beliefs about Indigenous Peoples Inequities (R – means reverse scoring)

| **Items** | **Strongly Disagree** | **2** | **3** | **Neutral** | **5** | **6** | **Strongly Agree** | **Do not know** |
| --- | --- | --- | --- | --- | --- | --- | --- | --- |
| Numerous policies put into place through the Indian Act over generations have contributed to the present-day health disparities affecting Indigenous peoples. |  |  |  |  |  |  |  |  |
| The negative effects of the Residential School System are a significant contributor to the health and social gaps that exist between Indigenous and non-Indigenous peoples today. |  |  |  |  |  |  |  |  |
| Indian Residential Schools were in the distant past so they probably don’t play a huge role in the health/social gaps that exist today between Indigenous and non-Indigenous Canadians (R) |  |  |  |  |  |  |  |  |
| The long-term effects of the Residential School System have been over-exaggerated in the media and/or society in general. (R) |  |  |  |  |  |  |  |  |
| The negative health effects of the Residential School System have been transferred from one generation to the next and contribute to present-day health inequities facing Indigenous peoples. |  |  |  |  |  |  |  |  |
| It seems unlikely that the Residential School System has negatively affected the well-being of the children and grandchildren of those who attended these schools. (R) |  |  |  |  |  |  |  |  |
| Indigenous peoples in some contexts do not receive equitable health services which contribute to ongoing health disparities. |  |  |  |  |  |  |  |  |
| Indigenous People in Canada have equal or more access to government-provided health care. (R) |  |  |  |  |  |  |  |  |
| Certain on-going government policies related to the provision of social services contribute to the ongoing health inequities facing the Indigenous peoples in Canada. |  |  |  |  |  |  |  |  |
| Indigenous Peoples in Canada receive the same or more funding for greater access to social and health services**.** (R) |  |  |  |  |  |  |  |  |
| There is no relationship between the social determinants of health (e.g., housing, income, education) and the historical and on-going health and social gaps between Indigenous and non-Indigenous peoples (R). |  |  |  |  |  |  |  |  |
| Differences between Indigenous and non-Indigenous peoples in key social determinants of health such as income and education play a significant role in contributing to health and social inequities between these groups |  |  |  |  |  |  |  |  |

Modified items assessing Blaming attitudes towards Indigenous Peoples

| **Items** | **Strongly Disagree** | **2** | **3** | **Neutral** | **5** | **6** | **Strongly Agree** | **Do not know** |
| --- | --- | --- | --- | --- | --- | --- | --- | --- |
| Most of the health and social problems of Indigenous people are brought on by themselves. |  |  |  |  |  |  |  |  |
| The root causes contributing to the health and social inequities facing Indigenous peoples today have been out of their control. (R) |  |  |  |  |  |  |  |  |
| Indigenous Peoples in Canada face unique historical, cultural, and social determinants of health associated with colonization that has affected their well-being. (R) |  |  |  |  |  |  |  |  |
| Many Indigenous peoples face unique obstacles that influence their opportunities to be successful and prosperous. (R) |  |  |  |  |  |  |  |  |
| Unfair and discriminatory social structures and institutions continue to promote “White” and “Settler” privilege at the expense of Indigenous peoples. (R) |  |  |  |  |  |  |  |  |
| Many other ethno-racial groups overcame racism and worked their way up so Indigenous peoples should be able to do the same. |  |  |  |  |  |  |  |  |
| If Indigenous people worked harder, they could have an equal chance to be successful and well-off. |  |  |  |  |  |  |  |  |
| Everyone has equal opportunity, so this so-called “White” and/or “Settler” privilege is really an excuse for the health inequities facing Indigenous peoples**.** |  |  |  |  |  |  |  |  |

Support for government responsibility and policies and actions to reduce inequalities

| **Items** | **Strongly Disagree** | **2** | **3** | **Neutral** | **5** | **6** | **Strongly Agree** |
| --- | --- | --- | --- | --- | --- | --- | --- |
| The federal government is spending too much on improving the living conditions of Indigenous Peoples. (R) |  |  |  |  |  |  |  |
| The federal government is not putting enough funding towards improving the health and well-being of Indigenous Peoples. |  |  |  |  |  |  |  |
| Social policies for Indigenous Peoples such as affirmative action should not be instituted because they discriminate unfairly against others. (R) |  |  |  |  |  |  |  |
| Programs such as affirmative action should be instituted in medical schools and other contexts to help create equity for Indigenous Peoples. |  |  |  |  |  |  |  |
| Indigenous Peoples should be treated like all Canadians and should not have any special benefits or rights to land or to hunt/fish. (r) |  |  |  |  |  |  |  |
| It is not the responsibility of our governments to provide special attention to reducing the health and social inequities facing Indigenous Peoples. (R) |  |  |  |  |  |  |  |
| The inherent rights of Indigenous Peoples in Canada should be upheld by our governments and our country. |  |  |  |  |  |  |  |
| All levels of government have an obligation to work with Indigenous Peoples to reduce health and social inequities. |  |  |  |  |  |  |  |
| It is the responsibility of all levels of governments to support the improvement of Indigenous Peoples’ health and social conditions. |  |  |  |  |  |  |  |

Perceived responsibility as a future healthcare provider

| **Items** | **Strongly Disagree** | **2** | **3** | **Neutral** | **5** | **6** | **Strongly Agree** |
| --- | --- | --- | --- | --- | --- | --- | --- |
| Please indicate the degree to which you agree or disagree with the following statements about what you consider to be your responsibility when you are a **health care provider:** | | | | | | | |
| I have a social responsibility to work with Indigenous Peoples to improve their social and health conditions. |  |  |  |  |  |  |  |
| It is important for me to advocate with Indigenous Peoples for the improvement of their health and well-being. |  |  |  |  |  |  |  |
| I do not have a social responsibility to consider the unique factors that might influence the experiences of Indigenous patients seeking health care. (R) |  |  |  |  |  |  |  |
| My mandate as a health professional does not include attention to the unique factors that may affect Indigenous peoples and is instead focused on providing equal care to all patients. (R) |  |  |  |  |  |  |  |
