# Supplementary material for: Beliefs around the causes of inequities and intergroup attitudes among health professional students before and after a course related to Indigenous Peoples and colonialism
Source: BMC Med Educ. 2023 Apr 22;23:277. doi: 10.1186/s12909-023-04248-7 (PMC10121421; doi:10.1186/s12909-023-04248-7)
Supplement: Supplementary file 2 — Additional file 2. Development of the Causal beliefs aboutIndigenous Peoples inequities Measure. [file 12909_2023_4248_MOESM2_ESM.docx]

Additional File 2

Development of the Causal beliefs about Indigenous Peoples inequities Measure

| **2019 Survey Items** | **2020 Survey Items** | **2022 Survey Items** |
| --- | --- | --- |
| The negative health effects of the residential school system have been transferred from one generation to the next and contribute to present-day health/social gaps facing Indigenous peoples | The negative effects of the Indian Residential School System are a significant contributor to the health and social gaps that exist between Indigenous and non-Indigenous peoples today | The negative effects of the Indian Residential School System are a significant contributor to the health and social gaps that exist between Indigenous and non-Indigenous peoples today |
| It seems unlikely that the residential school system has negatively affected the well-being of the children and grandchildren of those who attended these schools and is still a significant cause of health/social gaps relative to the non-Indigenous population. (R) | It is unlikely that the Indian Residential School System has negatively affected the well-being of the children and grandchildren of those who attended and doesn’t play a significant role in the well-being of Indigenous peoples today (R) | It seems unlikely that the Indian Residential School System has negatively affected the well-being of the children and grandchildren of those who attended (R) |
|  | Indian Residential Schools were in the distant past so they probably don’t play a huge role in the health/social gaps that exist today between Indigenous and non-Indigenous Canadians (R) | Indian Residential Schools were in the distant past, so they probably don’t play a huge role in the health/social gaps that exist today between Indigenous and non-Indigenous Canadians (R) |
|  | The negative effects of the Indian Residential School System have been transferred from one generation to the next and contribute to ongoing gaps in health/social outcomes between Indigenous and non-Indigenous peoples | The negative effects of the Residential School System have been transferred from one generation to the next and contribute to present-day health inequities facing Indigenous Peoples |
| The numerous harmful aspects of colonization that occurred before and after the Indian Residential School system over generations continues to contribute to health and social gaps between Indigenous and non-Indigenous peoples today. | Numerous aspects of colonization that occurred before, during and after the Indian Residential School system (e.g., Indian Act, forced relocations, Sixties Scoop) have had negative intergenerational effects and contribute to gaps in health/social outcomes | Numerous policies put into place through the Indian Act over generations have contributed to present-day health disparities affecting Indigenous Peoples |
| The long-term effects of the residential school system have been over-exaggerated in the media and/or society in general (R) | The long-term effects of the residential school system have been over-exaggerated in the media and/or society in general (R) | The long-term effects of the Residential School System have been over-exaggerated in the media (R) |
| Indigenous peoples in some contexts do not receive equitable health services which contributes to ongoing health/social gaps | Indigenous peoples in some contexts have reduced access to certain health/social services relative to non-Indigenous Canadians, which contributes to ongoing health/social gaps | Indigenous Peoples in some contexts do not receive equitable health services which contribute to ongoing health disparities |
| The Indigenous population in Canada have equal access to government provided health care. | Indigenous peoples in Canada have equal or more access to government provided health care and social services compared to non-Indigenous Canadians **(R)** | Indigenous peoples in Canada have equal or more access to government provided health care **(R)** |
| Government policies related to the provision of social services contribute to the ongoing health inequities facing the Indigenous peoples in Canada. | Certain ongoing government policies related to the provision of social/health services for Indigenous peoples contributes to the ongoing health/social gaps between Indigenous and non-Indigenous peoples | Certain ongoing government policies related to the provision of social services contribute to the ongoing health inequities facing Indigenous Peoples in Canada |
| Indigenous peoples in Canada receive the same amount or more funding for social and health services relative to non-Indigenous Canadians (R) | Indigenous peoples in Canada receive the same amount or more funding for social and health services relative to non-Indigenous Canadians (R) | Indigenous peoples in Canada receive the same amount or more funding for greater access to social and health services (R) |
|  | Differences between Indigenous and non-Indigenous peoples in key social determinants of health such as income and education play a significant role in contributing to health and social inequities between these groups | Differences between Indigenous and non-Indigenous peoples in key social determinants of health such as income and education play a significant role in contributing to health and social inequities between these groups |
|  | There is no relationship between the social determinants of health and the historical and on-going health and social gaps between Indigenous and non-Indigenous peoples **(R)** | There is no relationship between the social determinants of health (e.g., housing, income, education) and the historical and on-going health and social gaps between Indigenous and non-Indigenous peoples **(R).** |
